# Evolutionary mechanisms underlying bacterial adaptation to the plant environment

**DOI:** 10.1093/femsre/fuag005

**Published:** 2026-02-11

**Authors:** Zaki Saati-Santamaría, Daniel Pérez-Mendoza, Muhammad Khashi u Rahman, Bruna Fernanda Silva de Sousa, Maria del Carmen Montero-Calasanz, Luis Rey, Sonali Roy, Juan Sanjuán, Paula García-Fraile

**Affiliations:** Institute for Agribiotechnology Research (CIALE), Villamayor 37185 Salamanca, Spain; Unidad de Excelencia Producción, Agrícola y Medioambiente (AGRIENVIRONMENT), Universidad de Salamanca, 37185 Salamanca, Spain; Departamento de Microbiología y Genética, Universidad de Salamanca, 37007 Salamanca, Spain; Laboratory of Fungal Genetics and Metabolism, Institute of Microbiology of the Czech Academy of Sciences, Vídeňská 1083, 142 20 Prague, Czech Republic; Department of Soil and Plant Microbiology, Estación Experimental del Zaidín, CSIC, 18008 Granada, Spain; Institute for Agribiotechnology Research (CIALE), Villamayor 37185 Salamanca, Spain; Unidad de Excelencia Producción, Agrícola y Medioambiente (AGRIENVIRONMENT), Universidad de Salamanca, 37185 Salamanca, Spain; Departamento de Microbiología y Genética, Universidad de Salamanca, 37007 Salamanca, Spain; Centro de Biotecnología y Genómica de Plantas, Universidad Politécnica de Madrid (UPM)-Instituto Nacional de Investigación y Tecnología Agraria y Alimentaria (INIA/CSIC), Campus de Montegancedo, 28223 Pozuelo de Alarcón, Madrid, Spain; IFAPA Las Torres-Andalusian Institute of Agricultural and Fisheries Research and Training, Junta de Andalucía, Cra. Sevilla-Cazalla, km 12.2. 41200 Alcalá del Río, Seville, Spain; Centro de Biotecnología y Genómica de Plantas, Universidad Politécnica de Madrid (UPM)-Instituto Nacional de Investigación y Tecnología Agraria y Alimentaria (INIA/CSIC), Campus de Montegancedo, 28223 Pozuelo de Alarcón, Madrid, Spain; Departamento de Biotecnología y Biología Vegetal, ETSI Agronómica, Alimentaria y de Biostemas, Universidad Politécnica de Madrid, 28040 Madrid, Spain; College of Agriculture, Tennessee State University, 37209 Nashville, Tennessee, United States; Department of Soil and Plant Microbiology, Estación Experimental del Zaidín, CSIC, 18008 Granada, Spain; Institute for Agribiotechnology Research (CIALE), Villamayor 37185 Salamanca, Spain; Unidad de Excelencia Producción, Agrícola y Medioambiente (AGRIENVIRONMENT), Universidad de Salamanca, 37185 Salamanca, Spain; Departamento de Microbiología y Genética, Universidad de Salamanca, 37007 Salamanca, Spain; Associated Research Unit of Plant-Microorganism Interaction, USAL-CSIC (IRNASA), 37008 Salamanca, Spain

**Keywords:** bacterial adaptation, plant-microbe interactions, horizontal gene transfer (hgt), microbial ecology, evolutionary mechanisms, evolutionary ecology

## Abstract

Plants and bacteria have coevolved over hundreds of millions of years, forming complex associations ranging from mutualism to pathogenicity that are essential for plant survival and ecosystem function. Bacterial adaptation to plant environments involves dynamic evolutionary mechanisms including horizontal gene transfer, gene regulation, and metabolic specialization, enabling bacteria to persist and specialize within diverse plant-associated niches. Here we review how evolutionary forces such as selection, drift, and gene flow shape bacterial genomes, regulatory networks, and ecological strategies in response to plant-imposed pressures, underpinning both beneficial and pathogenic lifestyles. Understanding these processes provides a unified evolutionary framework for bacterial adaptation to plants and highlights their implications for sustainable agriculture and microbiome-based innovations.

## Teaser

This review explores how evolutionary mechanisms—from gene transfer to regulatory rewiring—enable bacteria to adapt to diverse plant niches, shaping interactions that span mutualism to disease.

## Introduction

The intricate relationship between plants and bacteria has been shaped by hundreds of millions of years of coevolution (Huang and Wang 2025). These long-standing associations encompass a wide spectrum of interactions, from mutualistic symbioses to antagonistic pathogenic relationships, forming an essential component of plant survival, ecosystem functioning, and agricultural productivity (Fields and Friman 2022, Mesny et al. 2023).

Plant-associated bacteria (PAB) facilitate nutrient acquisition through nitrogen fixation, phosphate solubilization, or siderophore-mediated iron uptake, directly contribute to plant metabolism (Velázquez et al. 2010, Chepsergon et al. 2023, Mishra et al. 2023, Compant et al. 2025). Others protect plants from biotic and abiotic stress, producing phytohormones, antimicrobial compounds, or inducing systemic resistance (Liu et al. 2024a, Xiang et al. 2025, Conrath 2025). Some even influence crop nutritional quality and resilience (Flores-Félix et al. 2015, 2018, Jiménez-Gómez et al. 2021, Roca-Couso et al. 2025). Conversely, pathogenic bacteria exploit plant resources through highly specialized virulence strategies (Barak and Schroeder 2012, Sacristán et al. 2021, Vailleau and Genin 2023). Between these two extremes lies a continuum of ecological strategies where bacterial lineages can establish commensal or transient associations by colonizing plant surfaces or tissues without clear beneficial or harmful effects (Hirsch 2004, Zhang et al. 2025a).

These relationships, though often overlooked, may serve as evolutionary intermediates and reservoirs of genetic and functional diversity, from which both symbionts and pathogens can emerge. These diverse lifestyles within the plant holobiont underscores the remarkable adaptive capacity of bacteria whose evolution is continuously shaped by the competitive and selective constraints imposed by host environment (Jwa et al. 2017, Li et al. 2021a).

In this review, we focus on the evolutionary mechanisms that enable bacteria to persist, specialize, and evolve within plant-associated habitats. Rather than summarizing the known molecular functions of plant–bacteria interactions, we examine how evolutionary forces such as selection, drift, and gene flow, shape bacterial genomes, regulatory networks, and ecological strategies in response to plant-imposed pressures. We discuss how these processes give rise to functional diversification, niche specialization, and co-metabolic integration, and how they underpin both mutualistic and pathogenic lifestyles. This review aims to provide a unified evolutionary framework for understanding bacterial adaptation to plants and to highlight its implications for sustainable agriculture and microbiome-based innovation.

## Evolutionary mechanisms and pressures underlying bacterial adaptation

Understanding the mechanisms that sustain evolutionary dynamism between microbes and their hosts requires examining the genetic and ecological processes that generate and maintain bacterial diversity within plant environments. Bacterial genome evolution is underpinned by diverse mechanisms that continually reshape the genetic landscape of PAB. Horizontal gene transfer (HGT) is a primary driver, enabling the acquisition of novel traits and genetic information via mobile genetic elements (MGEs) such as plasmids, genomic islands, transposons, and bacteriophages (Arnold et al. 2022, Saati-Santamaría et al. 2025). These MGEs are often interconnected, with plasmids acting as vehicles for transposons and integrons—genetic platforms that capture, rearrange, and express gene cassettes via site-specific recombination (Mazel 2006, Kieffer et al. 2025)—which can subsequently integrate into the chromosome and promote adaptation (Rodríguez-Beltrán et al. 2021). In plant-associated environments, such as the soil and rhizosphere, these processes are particularly active, as dense and diverse microbial communities constitute rich reservoirs of genes and MGEs that can be exchanged among populations, providing rhizobacteria with the genetic tools to cope with environmental stresses and plant defences (Vanga et al. 2015, De Assis et al. 2022).

PAB often carry key genes for host interaction on plasmids, pathogenicity islands, or symbiotic islands that bear signatures of past HGT events. One of the best-known examples involves rhizobial megaplasmids, often exceeding 1 Mb and functioning as secondary chromosomes or chromids. These large replicons encode essential symbiotic genes for nodulation and nitrogen fixation (Zahran 2017), and are frequently transferred among rhizobial populations, suggesting that symbiosis genes are part of a dynamic MGE-driven ecology. Such acquisitions can convert a non-nodulating *Mesorhizobium* into a fully capable symbiont (Wardell et al. 2022). Notably, large plasmids are not exclusive to plant beneficial bacteria. Members of the *Ralstonia solanacearum* species complex also harbour megaplasmids of >2 Mb, which carry pathogenicity-related genes (i.e. *hrp* genes) (Salanoubat et al. 2002). Beyond symbiosis and virulence, recent work has revealed that many PAB megaplasmids encode secondary metabolites (also known as specialized metabolites) via plasmid-borne Biosynthetic Gene Clusters. These include ribosomally synthesized and post-translationally modified peptides, siderophores, quorum-sensing molecules like N-Acyl homoserine lactones (AHLs), and cryptic nonribosomal peptide synthetase or polyketide synthase systems (Saati-Santamaría 2023). Such plasmid-encoded metabolites might modulate plant–microbe interactions by influencing signalling, nutrient exchange, or microbial competition (Loh et al. 2002, Dror et al. 2020).

Bacteriophages, genomic islands, integrons, and transposons also contribute to genome plasticity in PAB. Recent evidence shows that prophages in the phyllosphere can mediate the horizontal transfer of virulence effectors, such as hopAR1, across distant *Pseudomonas syringae* phylogroups, highlighting their potential to accelerate the evolution of pathogenic traits *in situ* (Hulin et al. 2023). Temperate phages may facilitate gene flow via lysogeny and transduction in densely packed root environments, where spatial proximity enhances the likelihood of HGT (de Sousa et al. 2023). Recent findings suggest that integrons and phage satellites—small mobile genetic elements that depend on helper phages for their replication and mobilization, and can modulate phage infection outcomes while carrying adaptive genes (Penadés et al. 2025)—are widespread in plant microbiomes, with potential implications for both mutualistic and pathogenic lifestyles (Ghaly et al. 2024). These MGEs provide a substrate for functional innovation, for instance by harbouring effector genes delivered by the Type III Secretion System (T3SS), often located on pathogenicity or symbiotic islands (Tan et al. 2019). Comparative genomics *of P. syringae* indicates that effector genes are among the most variable genome regions, frequently gained or lost as bacteria adapt to new hosts (Hulin et al. 2018, Djitro et al. 2022), enabling strains to rapidly acquire “weapons” to overcome plant defenses. In parallel, integrons are particularly abundant in *Pseudomonas, Burkholderia*, and *Xanthomonas*, with rhizosphere samples enriched in gene cassettes for metabolite transport and catabolism, likely facilitating utilization of diverse root exudates and resistance to plant or microbial antimicrobials (Ghaly et al. 2024).

Horizontal transfer is more likely between abundant taxa sharing moderate phylogenetic similarity, a pattern especially marked in plant-associated environments (Dmitrijeva et al. 2024), where spatial structure and host-derived signals may foster gene exchange (de Sousa et al. 2023). On the contrary, while plant microbiomes can promote HGT by providing dense and structured habitats that facilitate cell-to-cell contact, other studies suggest a parallel trend of mobilome reduction in PAB, especially in aerial compartments (Bograd et al. 2025). This apparent contradiction highlights the dual nature of plant-associated environments: they can act both as catalysts and as filters of horizontal gene exchange, depending on the niche, microbial lifestyle, and selective constraints imposed by the host. This dynamic landscape of MGE-driven gene flow extends even further when considering cross-kingdom HGT—for instance, the acquisition of auxin biosynthesis genes from bacteria to plants, or plant-derived genes involved in carbohydrate metabolism found in bacteria—illustrating the far-reaching functional consequences of gene flow across domains (Haimlich et al. 2024). Moreover, it is important to note that while HGT often enhances host adaptability, many transfers are also driven by the intrinsic evolutionary strategies of MGEs themselves, which can persist and spread even when their maintenance imposes a cost on the host (Wardell et al. 2022, Haudiquet et al. 2022, Lang et al. 2025).

Beyond HGT, other mechanisms such as gene duplication, point mutations, and homologous recombination also shape bacterial adaptation to plant-associated lifestyles. Gene duplication and amplification generate genetic redundancy and raw material for functional divergence (Álvarez-Lugo and Becerra 2021, Sudol et al. 2024, Fuentes-Ugarte et al. 2025). Point mutations, including single nucleotide polymorphisms, insertions, and deletions, are a primary source of variation and have been shown to drive rapid adaptation in experimental evolution settings. For instance, a *Pseudomonas protegens* strain evolved from antagonist to mutualist lifestyle via mutations in the *gacS*/*gacA* regulatory system, enhancing rhizosphere colonization and tolerance to plant antimicrobials (Li et al. 2021b). Similarly, short-term evolution of *Pseudomonas bijieensis* 2P24 over 297 generations in the wheat rhizosphere led to diversification into genotypes with improved colonization ability and competitive fitness, driven by recurrent mutations in the flagellar regulator *fleN* that modulated motility and surface attachment strategies (Li et al. 2025). Independent mutations in *efpR* improved the fitness of *R. solanacearum* on novel hosts (Guidot et al. 2014), while alterations in a virulence regulator (*hrpG*) or in a T3SS component (*hrcV*) enabled symbiotic behaviour after acquiring symbiotic plasmids (Marchetti et al. 2010). Homologous recombination further contributes to genome plasticity by facilitating the gain or loss of ecologically relevant *loci*. In the *Pseudomonas fluorescens* complex, such recombination mediates convergent acquisition of genomic islands controlling lipopeptide biosynthesis or quorum sensing, underpinning transitions between commensalism and pathogenesis (Melnyk et al. 2019). Altogether, these mechanisms support the dynamic evolution of PAB by enabling both fine-scale genetic tuning and large-scale functional shifts.

In addition to genetic changes, bacteria can rapidly adapt to plant environments through regulatory mechanisms that do not involve changes in DNA sequence. In plant-associated settings, bacteria perceive and respond to host-derived chemical cues, such as sugars, organic acids, amino acids, and secondary metabolites present in root exudates, which act as signals that modulate transcriptional programs involved in metabolism, motility, chemotaxis, and colonization (Pankievicz et al. 2016, Liao et al. 2019, Vanier et al. 2023, Saati-Santamaría et al. 2026). These signals are integrated through regulatory networks involving transcription factors, two-component systems, and global regulators, allowing bacteria to adjust their physiology to specific plant hosts or compartments (discussed in depth in the following sections). In parallel, epigenetic regulation—most notably DNA methylation mediated by restriction–modification systems or orphan methyltransferases—can influence gene expression patterns in a reversible or semi-heritable manner, generating phenotypic heterogeneity within clonal populations (DiCenzo et al. 2022, Gopalan-Nair et al. 2024, Babińska-Wensierska et al. 2025). These regulatory strategies complement genetic mechanisms by allowing fine-tuned, context-dependent responses that enhance bacterial adaptation in dynamic and spatially structured plant-associated niches.

These mechanisms generate variation within PAB populations, but its fate depends on the evolutionary forces at play. The balance between natural selection and genetic drift in microbial populations is shaped by effective population size (N_e_)—size of an idealized population that would experience the same strength of genetic drift as the observed population—(Wright 1931, Charlesworth 2009, Wang et al. 2016), and which may vary between plant compartments. In the rhizosphere, large populations favour natural selection. Conversely, the endosphere imposes ecological filtering and colonization bottlenecks that reduce N_e_, increasing the influence of genetic drift. While supported by other host-associated systems (Didelot et al. 2016, Sheppard et al. 2018), such as viral evolution in resistant plant genotypes (Tamisier et al. 2024), direct empirical evidence in plant microbiomes remains limited. For instance, Noda-García et al. (2019) examined the dynamics of allele fixation in soil and root environments, revealing that both drift and selection act on these niches. However, the study was not designed to disentangle these forces in planta, nor to assess how variation in N_e_ across plant compartments might influence evolutionary trajectories. In any case, such contrasting population dynamics likely modulate the tempo and mode of evolutionary processes described above. As a result, generalist rhizosphere colonizers may evolve broad metabolic plasticity via HGT transfer and adaptive mutations, while endophytic specialists may be shaped by stronger host-imposed filtering and selection, leading to highly specific traits for host interaction and nutrient acquisition. In systems like rhizobia, where symbiosis genes are often carried on mobile elements (e.g. pSyms or ICEsyms), spatial and temporal heterogeneity in host presence may further favour the maintenance of these elements through horizontal transfer and bet-hedging strategies, particularly in rhizosphere populations with large N_e_ (Wardell et al. 2022). Conversely, the reduced population size and repeated bottlenecks in planta likely weaken the efficacy of selection, allowing stochastic processes to dominate. Under such conditions, costly mobile elements may persist or be lost largely by drift, unless their contribution to host adaptation is sufficiently large to overcome demographic constraints (Charlesworth 2009). Still, the maintenance of such genes does not always require a current selective advantage. Genes embedded in mobile elements may persist transiently by hitchhiking with successful genomic backgrounds, benefiting from context-dependent associations with strains or traits under positive selection (Douglas and Shapiro 2021, Haudiquet et al. 2022). This process may facilitate their reactivation under favourable environmental or host conditions.

Ultimately, the evolutionary fate of these mobile and adaptive traits depends on the ecological opportunities available within plant-associated habitats, particularly the capacity of bacteria to exploit host-derived resources. The selective advantage conferred by metabolic versatility provides the foundation for the next stage of plant–microbe coevolution: specialization on plant-produced substrates.

## Microbial evolution to specificity of plant-derived resource utilization

**Figure 1:**
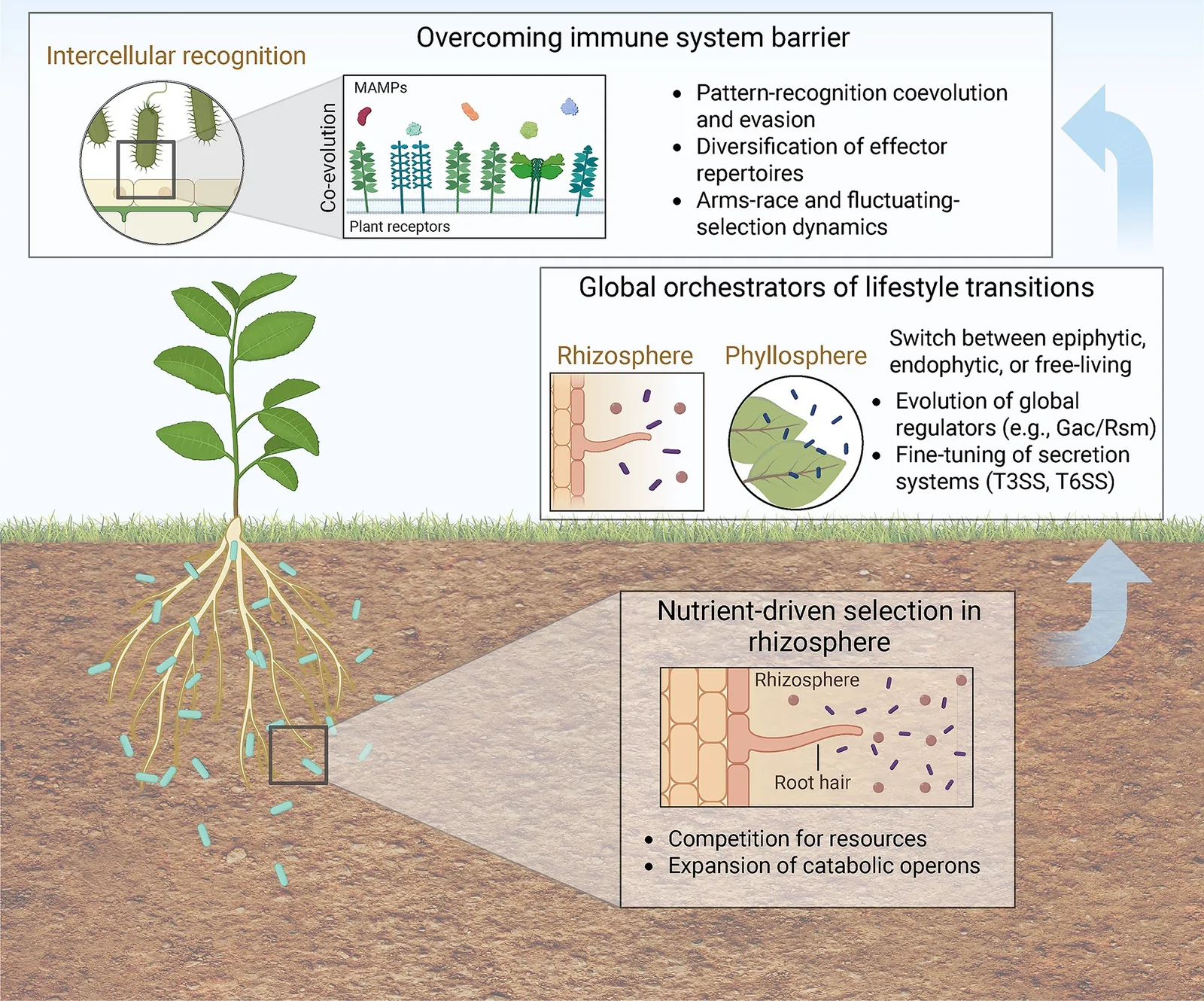
Evolutionary mechanisms underlying plant–bacteria interactions across spatial compartments. Bacterial colonization in the rhizosphere is shaped by strong selective pressures imposed by root-derived nutrients and intense microbial competition. As bacteria transition across plant-associated niches, including the rhizosphere and phyllosphere, they rely on global lifestyle orchestrators (e.g. Gac/Rsm systems and other regulatory hubs) that coordinate shifts in metabolism, motility, and stress responses to ensure niche adaptability. At the host interface, successful plant colonizers must overcome immune recognition, leading to the diversification of MAMP structures, effector repertoires, and immune-evasion strategies. These antagonistic pressures drive dynamic evolutionary outcomes, including arms-race and fluctuating selection processes. Each panel corresponds to a major thematic section discussed in this review. Image created with BioRender.com.

Building upon these genetic and regulatory foundations, the transition from free-living to plant-associated lifestyles is often initiated by the ability to exploit plant-derived substrates (Wiesmann et al. 2023) (Fig. 1). The rhizosphere, enriched in root exudates that can represent up to 40% of photosynthetically fixed carbon (Paterson et al. 1996, Pausch and Kuzyakov 2018), constitutes a nutrient hotspot and a powerful selective filter shaping microbial evolution toward substrate specialization (Zhalnina et al. 2018, Preece and Peñuelas 2020). Similarly, the phyllosphere and other aerial tissues also present distinct chemical landscapes that influence microbial adaptation.

Microbial adaptation to the specific chemical profile of plant exudates is fundamentally a genetic process in which selection acts on mutations and HGT events (Good et al. 2025). Comparative and experimental genomics have revealed how exposure to plant-derived metabolites favours the retention or acquisition of catabolic functions, leading to resource-based diversification. For example, HGT can provide an immediate evolutionary advantage, as shown in *Burkholderia* species where the acquisition of a large plasmid confers a complete operon for the degradation of specific root exudates, enabling rapid adaptation to novel carbon sources in the rhizosphere (Zhu et al. 2011, Priya et al. 2024, Wang et al. 2024a). Across PAB, this adaptive trend often manifests as an enrichment in genes related to carbohydrate catabolism (López-Mondéjar et al. 2022, Saati-Santamaría et al. 2022, 2024, Segev et al. 2025). Experimental evidence supports this genetic–ecological link: in *Rhizobium leguminosarum* bv. *viciae*, mutants impaired in arabinose or protocatechuate catabolism (*araE* and *pcaM*, respectively) showed reduced competitiveness in the pea rhizosphere and diminished nodule infection efficiency when co-inoculated with the wild-type strain (García-Fraile et al. 2015). These results demonstrate that the capacity to catabolize specific plant-derived carbon sources is directly tied to rhizosphere fitness and successful symbiotic establishment.

Patterns of substrate specialization are also evident at the metagenomic scale. Surveys of the *Arabidopsis* root microbiome show consistent enrichment in bacterial chitinase genes (Eichfeld et al. 2024, Zhao et al. 2023), reflecting adaptation to utilize fungal cell wall components as accessible resources in root-associated environments. In contrast, the phyllosphere selects for genes enabling the degradation of plant structural polymers such as pectin and xylan (Costa et al. 2020, Li et al. 2024a), highlighting how distinct plant compartments generate divergent selective landscapes that drive genomic partitioning across bacterial taxa.

Beyond the acquisition of new catabolic capabilities, the evolutionary refinement of metabolic efficiency constitutes another layer of adaptation. In *Bacillus subtilis*, experimental evolution on different exudate-derived carbon sources leads to parallel but distinct adaptive trajectories: populations evolving on malate frequently upregulate the MaeA malic enzyme, optimizing central metabolism for energy yield, whereas glucose-adapted populations follow alternative genetic routes (Doan et al. 2003, Kleijn et al. 2010, Lerondel et al. 2006). Such fine-tuning exemplifies how adaptation extends beyond simple substrate use to encompass global metabolic reprogramming, an evolutionary process that fosters specialization and narrows ecological breadth.

Beyond individual metabolic optimization, microbes also evolve strategies to partition chemical niches by exploiting specific plant-derived substrates. Metabolic modelling and experimental studies show how different strains utilize distinct exudate components, promoting resource-based coexistence and shaping host-specific microbial assemblages. In axenic conditions, exposure to the root exudates of *Brachypodium distachyon* triggered distinct transcriptional programs in eight strains of the *P. fluorescens* group, directly linking strain-level niche differentiation to their accessory genomes (Mavrodi et al. 2021). Nearly half of the induced catabolic pathways were strain-specific, exemplified by the universal upregulation of a fructose phosphotransferase system (PTSFru) in most strains, contrasted with the selective induction of a myo-inositol degradation cluster in only a phylogenetically distinct subset.

Such metabolic specialization facilitates coexistence, allowing generalists to rapidly consume common exudates like glucose, while specialists exploit unique niches, for example *Rhizobium* sensing host-specific flavonoids or *Arthrobacter* degrading rare phenolics (Badri and Vivanco 2009, Huang et al. 2014). Similarly, *Sphingomonas* efficiently utilizes gluconate, whereas *Variovorax* specializes in scavenging N-acetylglucosamine (Beer et al. 2018, Sigurbjörnsdóttir and Vilhelmsson 2016). By partitioning resources in this way, microbial competition is reduced, fostering structured, host-specific communities and promoting adaptive radiation within PAB lineages driven by resource specialization.

A further example of resource-based niche differentiation is cross-feeding, a form of metabolic cooperation where one organism’s waste product becomes another’s substrate. Complex molecules are rarely fully mineralized by a single microbe; partial transformation releases intermediates that provide ecological opportunities for other specialists (Gu et al. 2025). A canonical example in the rhizosphere is the synergistic partnership between *Pseudomonas* and *Arthrobacter* in degrading the herbicide 2,4-dichlorophenoxyacetic acid (2,4-D), where the initial dechlorination performed by *Pseudomonas* yields chlorophenol, which can be subsequently mineralized by specialized *Arthrobacter* strains (Olaniran et al. 2017, Sahoo et al. 2022). Thus, cross-feeding reflects metabolic specialization and complementarity in substrate utilization, reinforcing niche partitioning among taxa and representing an evolutionary route toward metabolic interdependence and community-level adaptation

Plants can actively modulate the chemical composition of their exudates in response to biotic and abiotic stresses, creating dynamic selective pressures that further shape microbial adaptation. The “cry-for-help” hypothesis posits that plants under attack reconfigure their root exudate profile to selectively recruit beneficial microbes that benefit the host in combating stress (Rolfe et al. 2019). The classical example of evolutionary adaptation to this “call” is the chemotaxis systems of plant beneficial *Bacillus subtills* strains. These bacteria possess a chemoreceptor McpB, among others (Arnaouteli et al. 2021, Matilla and Krell 2024), which is sensitive to γ-aminobutyric acid (GABA)—a major plant stress metabolite that accumulates in the rhizosphere during insect herbivory (Yao et al. 2025). The evolution of this specific chemotaxis response allows *B. subtilis* to rapidly locate and colonize the root of plant under herbivory stress, where it can then establish a protective symbiotic relationship. The recruitment of protective rhizobacteria under pathogen attack (Liu et al. 2024b), herbivory (Yang et al. 2025), or abiotic stresses (Wang and Song 2022) demonstrates how microbial traits can become finely tuned to plant signalling. Certain plant-beneficial *Pseudomonas* strains carry conserved promoter elements upstream of their phenazine antibiotic biosynthesis clusters (Mavrodi et al. 2025). These are directly induced by certain root exudates, which can leak into the rhizosphere upon root damage or pathogen challenge. This creates a direct genetic link between the plant’s stress state and the activation of microbial antagonism, ensuring that the metabolically costly production of antibiotics is precisely timed to when it is most needed by the plant. Such microbial recruitment can influence the composition of the microbial community, favouring microbes possessing traits that provide direct benefits to the plant host (Arnaouteli et al. 2021, Matilla and Krell 2024, Mavrodi et al. 2025). The assembly of this community may be influenced by reciprocal selection, where plant exudates select for specific microbial functions and microbes evolve to detect and metabolize these compounds, thereby enhancing their fitness in the plant-defined environment.

## Bacterial orchestrators of bacterial lifestyle in plants

In particularly complex habitats such as the rhizosphere, plant interacting bacteria face the critical decision of whether to adopt a free-living, motile, and saprobic lifestyle or to engage in an aggregated lifestyle in close association with the host plant (Wiesmann et al. 2023) (Fig. 1). The outcome of this choice has profound implications for bacterial survival, competition, and ecological fitness within this advantageous niche. This ability not only confers ecological advantages but also represents a crucial factor in establishing effective interactions with eukaryotic hosts (Danhorn and Fuqua 2007).

To support such adaptive strategies, bacteria rely on sophisticated signal transduction systems that couple the perception of environmental cues to highly specific cellular and metabolic responses. These systems comprise diverse classes of sensory modules, including histidine kinases, membrane components of the sugar phosphotransferase system, methyl-accepting chemoreceptors, nucleotide cyclases and phosphodiesterases, extracytoplasmic function sigma factors, Ser/Thr/Tyr protein kinases and phosphoprotein phosphatases (Galperin 2005). Acting at different stages of the signal transduction cascade, these modules rarely transmit environmental signals, or first messengers, directly to cellular effectors. Instead, they frequently modulate the intracellular levels of small molecular intermediates collectively known as bacterial second messengers (Galperin 2005).

The incorporation of second messengers into bacterial signalling networks have provided key functional advantages during micro-organism’s evolution (Hengge et al. 2023). First, their small size allows rapid diffusion throughout the cytoplasm, ensuring fast responses. Second, their capacity for integration permits a single signalling system to process multiple environmental inputs. Third, their involvement enables amplification, allowing weak environmental signals to generate robust cellular responses. Finally, their intracellular concentrations are finely regulated by enzymatic synthesis and degradation. These enzymatic activities are often embedded within multidomain proteins that contain additional sensory domains (e.g. PAS, GAF, CheY) (Galperin 2004). In this way, the presence of environmental first messengers is transduced into a rapid increase or decrease in the intracellular levels of the corresponding second messenger. Subsequently, this second messenger can be recognized by a wide array of different cellular effectors, which execute their function in a fraction of the time required by other regulatory systems involving gene expression (transcription and translation), ensuring responses that are not only rapid but also highly precise and context-dependent (Rediers et al. 2005, Ceulemans et al. 2021). Together, these features highlight the central role of second messengers in enabling bacteria to thrive in fluctuating and competitive environments such as the rhizosphere.

In rhizobacteria, these signal transduction systems operate within highly integrated regulatory networks that control collective behaviours and adaptive responses. A paradigmatic example is quorum sensing, through which bacteria monitor cell density via diffusible signalling molecules and coordinate population-wide processes such as biofilm formation, virulence or symbiosis (Loh et al. 2002). Similarly, the Gac/Rsm regulatory system functions as a global post-transcriptional switch that modulates the expression of genes associated with secondary metabolism, motility, and host interactions (Martínez-Granero et al. 2012, Ferreiro and Gallegos 2021). Evolutionary studies show that adaptive mutations in *gacA*/*gacS* can shift bacterial lifestyles along the mutualism–pathogenicity continuum: for example, in *Pseudomonas protegens*, mutations in this two-component system enabled a rapid transition from plant-antagonistic to mutualistic behaviour within only a few plant generations, enhancing root colonization and tolerance to plant antimicrobials (Li et al. 2021b). Moreover, recent findings indicate that this regulatory network not only orchestrates endogenous genes but also integrates horizontally acquired elements. In *P. fluorescens*, closely related strains with contrasting lifestyles—a commensal carrying the DAPG biosynthetic cluster and a pathogenic strain harbouring a syringomycin/syringopeptin island—share a conserved GacA regulator. Loss of *gacA* in the pathogenic strain abolishes expression of the horizontally acquired toxin genes, reverting the strain toward a commensal phenotype, while cross-complementation of *gacA* alleles between strains restores regulation (Luo et al. 2025). These findings illustrate how the conserved Gac/Rsm regulatory core provides an evolutionary scaffold for the assimilation and conditional control of horizontally transferred traits, thereby enabling rapid shifts in ecological strategy without extensive rewiring of the underlying network.

Other two-component systems have evolved analogous adaptive functions. The CenK/CenR system in *Rhizobium etli* coordinates cell division and envelope homeostasis, and its disruption impairs both free-living growth and symbiotic nitrogen fixation, revealing the evolutionary coupling between cell cycle regulation and host adaptation (Banda et al. 2025). Likewise, the ExoS/ChvI and ChvG/ChvI systems are essential for the establishment of successful symbiosis in *Sinorhizobium meliloti* and virulence in *Agrobacterium tumefaciens*, highlighting how homologous regulators have diversified to support distinct host-associated lifestyles (Wiesmann et al. 2023).

At broader evolutionary scales, comparative and experimental studies reveal that adaptive diversification of transcriptional networks underlie bacterial niche specialization. In *R. solanacearum*, for instance, recurrent mutations in the regulatory gene *efpR* promote increased fitness on new hosts, while the emergence of *efpH*-mediated phenotypic heterogeneity allows coexisting metabolic and virulent phenotypes to persist under fluctuating plant environments—an adaptive strategy akin to bet-hedging (Guidot et al. 2014, Perrier et al. 2019, Gopalan-Nair et al. 2023). Similarly, the HrpG regulon in *Xanthomonas* shows extensive interspecific variation, suggesting that strain-specific rewiring of virulence regulation has facilitated adaptation to diverse hosts and ecological niches (Monnens et al. 2024).

Nucleotide-based second messengers (NSM) finely tune the transition between saprophytic, epiphytic and endophytic lifestyles (Pesavento and Hengge 2009). Together, these interconnected signalling modules exemplify the evolutionary refinement of bacterial regulatory networks that translate environmental information into coordinated, adaptive behaviours.

### Cyclic-di-GMP signal transduction in the rhizosphere: a molecular basis for adaptation, competitiveness, and evolution in PAB

Since its discovery as an activator of cellulose synthase (Ross et al 1986, 1990), bis-(3’,5’)-cyclic diguanosine monophosphate (cyclic diguanylate, c-di-GMP, cdG) has emerged as a universal bacterial messenger (Römling et al. 2013). The number of c-di-GMP-related proteins correlates with bacterial adaptive capacity (Galperin 2005, Galperin et al. 2010): bacteria in highly dynamic environments, such as the rhizosphere, encode a high number of such proteins—sometimes nearly 1% of their proteome—, whereas those living in more stable environments, such as obligate intracellular pathogens, possess few or none (Römling 2013 et al. 2013, Galperin 2005). In this context, the abundance of these and other sensory modules has been elegantly described by Galperin and colleagues as a quantitative means to define the versatility of bacteria in adapting to their surrounding environment, a concept they termed “Bacterial IQ” (Galperin et al. 2010, Table 1).

**Table 1:** Cyclic-di-GMP signalling proteins in different Rhizospheric plant-interacting bacteria.

Organism	Taxonomy ID	Proteome	Bacterial IQ	GGDEF	GGDEF + EAL	EAL	HD-GYP	Effectors	cdG	% cdG
*Agrobacterium fabrum* str. C58	176 299	5.402	95	16	13	1	1	3	34	0,63
*Azorhizobium caulinodans* ORS 571	438 753	4.717	116	24	10	4	1	3	42	0,89
*Azospirillum sp*. B510	137 722	6.309	nd	20	19	3	6	6	54	0,86
*Azotobacter vinelandii* DJ	322 710	5.050	nd	13	10	1	3	7	34	0,67
*Bradyrhizobium diazoefficiens* USDA 110	224 911	8.317	83	12	23	4	3	13	55	0,66
*Paraburkholderia phymatum* STM815	391 038	7.496	85	20	18	11	1	3	53	0,71
*Musicola paradisiaca* Ech703	579 405	3.970	nd	15	5	7	–	2	29	0,73
*Herbaspirillum seropedicae* SmR1	757 424	4.735	nd	27	16	4	11	3	61	1,29
*Pectobacterium atrosepticum* SCRI1043	218 491	4.472	100	13	4	7	1	3	28	0,63
*Pseudomonas protegens* Pf-5	220 664	6.137	106	21	19	10	4	8	62	1,01
*Pseudomonas putida* KT2440	160 488	5.350	109	19	17	3	2	10	51	0,95
*Pseudomonas stutzeri* A1501	379 731	4.128	132	19	15	3	2	11	50	1,21
*Pseudomonas syringae* pv. tomato str. DC3000	223 283	5.608	109	16	19	2	1	10	48	0,86
*Ralstonia pseudosolanacearum* GMI1000	267 608	5.116	100	12	10	4	2	5	33	0,65
*Rhizobium etli* CFN 42	347 834	5.963	98	14	21	–	1	7	43	0,72
*Rhizobium johnstonii* 3841	216 596	7.150	84	15	22	–	3	7	47	0,66
*Sinorhizobium meliloti* 1021	266 834	6.205	81	6	12	1	–	5	24	0,39
*Xanthomonas campestris* pv. campestris str. ATCC 33 913	190 485	4.181	123	21	10	4	3	5	43	1,03

Bacterial IQ and c-di-GMP (cdG) metabolizing and effector proteins are extracted from Galperin et al. (2010) and Römling et al. (2013), respectively. Effectors correspond to PilZ + other c-di-GMP-binding identified domains including MshEN, Transcriptional regulators, diverse Glycosiltransferases and other reporter receptors. cdG means the sum of all c-di-GMP metabolizing plus effector proteins. % cdG=(cdG/proteome)x100

**Figure 2:**
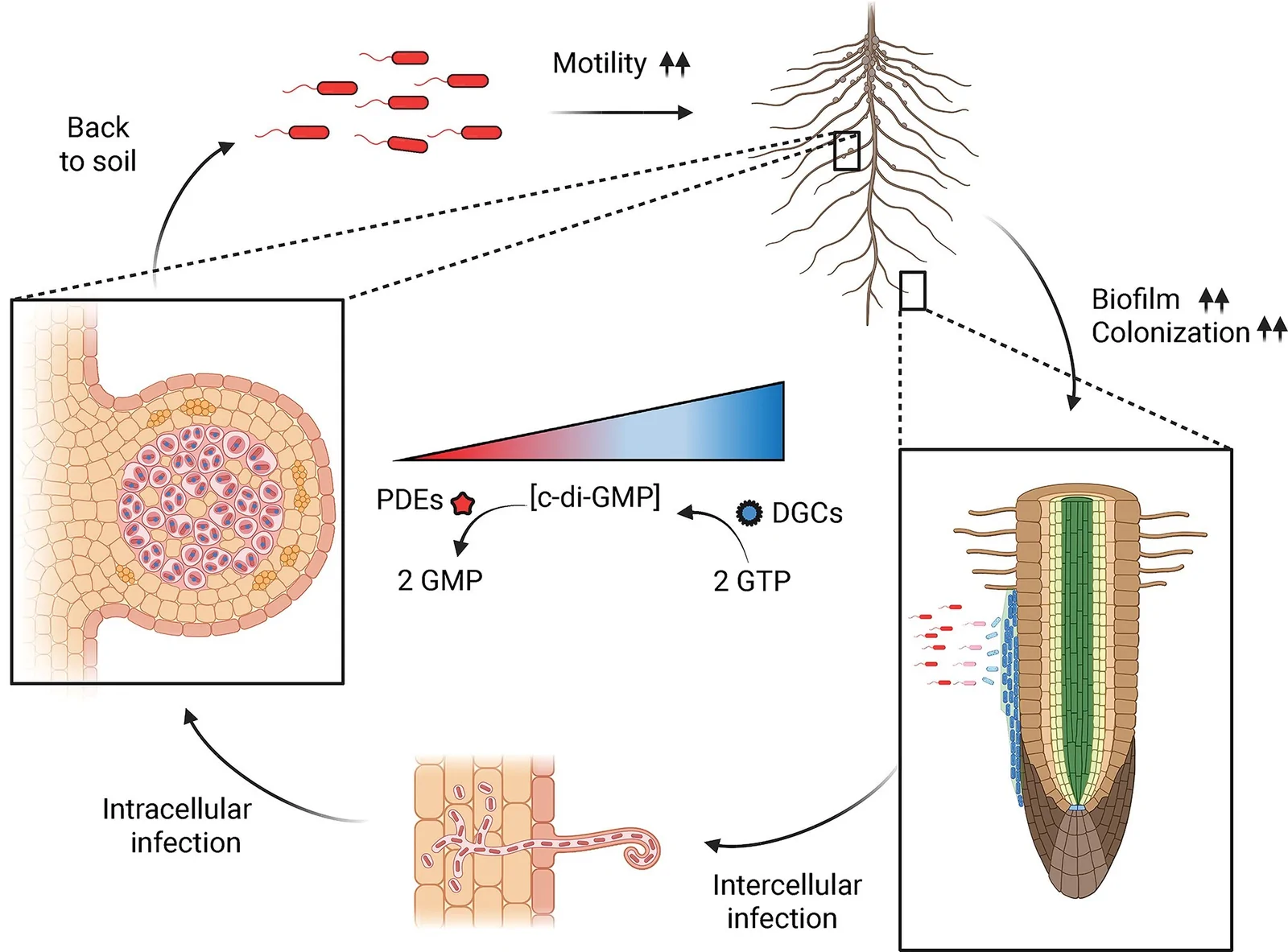
Relevance of c-di-GMP homeostasis in bacterial root infection. In saprophytic/planktonic stage, low c-di-GMP levels are needed in order to facilitate bacterial motility and chemotaxis towards root secreted nutritional compounds. After reaching the rhizosphere, adhesion to the rhizoplane and colonization of the root surfaces (as microcolonies or as biofilms) are promoted by increasing c-di-GMP levels. While most rhizosphere bacteria will not go beyond this stage, some can go further by colonizing the interior of the roots. This root infection is likely to be hindered by certain biofilm matrix components, therefore c-di-GMP levels must be decreased to favour intercellular infection. In the most intimate interactions, like the rhizobia-legume symbiosis, intracellular accomodation of bacteria involves cellular differentiations and functions that perhaps require a general reduction of c-di-GMP and a fine tunning of c-di-GMP production at precise subcellular locations. Image created with BioRender.com.

The molecular signalling mechanism of c-di-GMP is well characterized (Römling et al. 2013, Jenal et al. 2017, Hengge 2021). Briefly, c-di-GMP signalling systems typically consist of four principal components: (i) diguanylate cyclases (DGCs), which catalyze the synthesis of c-di-GMP from two GTP molecules; (ii) phosphodiesterases (PDEs), responsible for c-di-GMP degradation; (iii) c-di-GMP-binding effectors; and (iv) downstream target components that generate specific molecular outputs. The dynamic balance between synthesis and degradation ultimately defines intracellular c-di-GMP homeostasis (Fig. 2). Once synthesized, c-di-GMP has the ability to exert its regulatory action at (i) the transcriptional level—it can be recognized by and bind to transcriptional regulators, thereby modulating their affinity for specific promoters; (ii) the post-transcriptional level, interacting with mRNA molecules and altering their translational efficiency through mechanisms known as riboswitches; and (iii) the post-translational level, binding to enzymes, acting as an allosteric effector that modulates their activity. This multilayered regulation adds an additional degree of complexity to the systems in which it operates, providing even greater precision, a phenomenon that has been referred to as “sustained sensing” (Orr et al. 2016).

A substantial portion of our understanding of c-di-GMP regulation stems from studies on bacteria interacting with animal hosts (Obeng et al. 2023). Nevertheless, several discoveries during the last years highlight the central role of c-di-GMP homeostasis in the physiology and adaptive responses of PAB, including rhizosphere inhabitants, which alternate between a free-living motile lifestyle and a sessile state in close association with plant surfaces (Huang et al. 2024). These transitions are tightly governed by intracellular levels of c-di-GMP, which acts as a molecular switch coordinating motility, attachment, and biofilm formation. In response to root-derived chemical cues, low c-di-GMP concentrations promote flagellar motility and chemotaxis, facilitating colonization of new niches, whereas elevated c-di-GMP levels trigger surface attachment, EPS synthesis, and the establishment of biofilm communities that confer protection and stability in the rhizosphere (Huang et al. 2024).

Moreover, c-di-GMP not only orchestrates the transition between motile and sessile lifestyles but also represents an evolutionarily conserved mechanism that modulates key aspects of host interaction, such as adherence, secretion of virulence or symbiotic factors, and evasion of plant immunity (Pérez-Mendoza et al. 2014, Huang et al. 2024). For instance, recent studies have revealed that c-di-GMP signalling directly regulates the activity of the Type VI Secretion System (T6SS) (Aranda-Pérez et al. 2025). Originally characterized as a weapon for interbacterial competition (Basler and Mekalanos 2012 et al. 2012, Garin et al. 2025), which is now recognized as an important determinant of plant-associated lifestyles (Scouten et al. 2025). Early studies reported that T6SS activity in *Rhizobium* negatively affected symbiosis with peas (Bladergroen et al. 2003), whereas later work revealed positive roles for T6SS in *Rhizobium*–bean and *Rhizobium*–lupine interactions (Salinero-Lanzarote et al. 2019, Tighilt et al. 2022). These contrasting outcomes illustrate the evolutionary plasticity of the system. Although no T6SS effectors directly targeting plant cells have yet been identified, recent evidence suggests that some T6-dependent effectors may indirectly facilitate symbiosis; for example, a methyltransferase-domain effector that could modify membrane proteins to promote compatibility with lupine hosts (Tighilt et al. 2022). Thus, by intricately regulating the T6SS alongside other cellular pathways, c-di-GMP signalling integrates antagonistic and mutualistic strategies, fine-tuning bacterial behaviour to optimize plant–microbe interactions despite the still unresolved details of these processes.

While increased c-di-GMP levels favour colonization of the rhizosphere and the root surfaces, certain c-di-GMP activated circuits may become detrimental during intimate endosymbiotic stages (Pérez-Mendoza et al. 2014, Li et al. 2021c, Pérez-Mendoza et al. 2022). Consequently, bacteria have evolved mechanisms to silence specific c-di-GMP circuits. This evolutionary fine-tuning is particularly evident in rhizobia. In these systems, many diguanylate cyclases are transcriptionally silenced in bacteroids, maintaining low intracellular c-di-GMP levels compatible with nitrogen-fixing symbiosis. In soybean nodules induced by *Sinorhizobium fredii*, this silencing is mediated by the transcriptional regulator MucR (Li et al. 2021c), which simultaneously activates genes required for ion transport and nodule function (Jiao et al. 2016).

In free-living *S. meliloti*, MucR coordinates a broader adaptive program, enhancing root colonization and nodule initiation while repressing genes expressed during later stages of the symbiosis (Mueller and González 2011). The evolutionary conservation of the Ros/MucR family across α-proteobacteria reflects its deep integration into regulatory circuits controlling host interaction. These Zn-finger regulators not only bind AT-rich regions of DNA but also bridge and condense the chromosome (Chaves-Sanjuan et al. 2024), properties shared with H-NS-like proteins. Such structural and functional convergence suggests that Ros/MucR proteins have been repeatedly co-opted during evolution as “xenogeneic silencers”—regulators that modulate the expression of horizontally acquired genes (e.g. symbiosis plasmids and islands) until their expression becomes selectively advantageous (Baglivo et al. 2025).

From an evolutionary standpoint, this mechanism represents an elegant solution to the tension between innovation and stability: it allows the acquisition of foreign, potentially adaptive genes while minimizing their immediate fitness cost. Indeed, MucR not only facilitates the integration of AT-rich genomic regions (Shi et al. 2022) but also represses their premature expression (Jiao et al. 2022), enabling bacteria to explore new genomic space without compromising homeostasis. Despite limited sequence conservation, MucR shares additional mechanistic features with H-NS regulators beyond DNA binding and bridging (Chaves-Sanjuan et al. 2024). Both protein families are now known to interface with c-di-GMP signalling networks, providing a molecular link between environmental sensing and regulatory silencing. In *Salmonella*, c-di-GMP directly binds H-NS, inhibiting its DNA-binding activity and derepressing previously silenced genes (Li et al. 2023). Similarly, in α-proteobacteria, c-di-GMP interferes with the DNA-bridging function of MucR, acting as an anti-silencing cue that modulates the expression of symbiosis-related *loci* (Liu et al. 2025).

These findings reveal c-di-GMP as a central evolutionary integrator, connecting environmental perception, regulatory innovation, and genome plasticity. By coupling the repression and reactivation of horizontally acquired genes to the metabolic state of the cell, bacteria ensure that adaptive traits are expressed precisely when ecological or host-derived cues make them advantageous, consolidating the molecular basis for plant-associated specialization.

## Bacterial adaptation to plant defence mechanisms

Once bacteria have successfully transitioned from a free-living to a plant-associated lifestyle, a new adaptive challenge emerges: survival within the immunologically active plant environment. The rhizosphere and endosphere are not passive habitats but selective landscapes shaped by plant defense mechanisms that continuously impose pressure on microbial populations (Han 2019, Teixeira et al. 2019). In this context, bacterial evolution is further driven by the need to persist, evade, or modulate host immunity, processes that have given rise to intricate molecular strategies of immune interference, mimicry, and tolerance (Fig. 1).

### The plant immune system as a selective landscape

The interaction between plants and PAB is a dynamic co-evolutionary process, with the plant innate immune system acting as a powerful selective force (McCann et al. 2012, Han 2019, Miller et. al. 2017). Plant immunity is a complex, multi-layered system designed to sense and respond to microbial threats or engage with beneficial or commensal microbes, determining the outcome of host-microbe interactions. The first line of defence is triggered by the perception of conserved microbial-associated molecular patterns (MAMPs) via plasma-membrane-localized pattern recognition receptors (PRRs). MAMPs, which are essential for microbial life, include fungal chitin, bacterial flagellin, the translational elongation factor Tu (EF-Tu), peptidoglycan (PGN), and lipopolysaccharides (LPS) (DeFalco and Zipfel 2021, Zhang et al. 2024). These molecules are recognized by PRRs on the plant cell surface, including receptor-like kinases such as FLAGELLIN-SENSING 2 (FLS2) and ELONGATION FACTOR RECEPTOR (EFR), as well as LysM-domain-containing receptors that bind PGN or chitin-like motifs (DeFalco and Zipfel 2021, Zhang et al. 2024). Upon detection, plants trigger a basal immune response known as PAMP-triggered immunity (PTI), which involves transcriptional reprogramming, callose deposition in the cell wall, and the production of reactive oxygen species (ROS), ion influx, and biosynthesis of plant defense hormones, collectively restricting microbial colonization (Peng et al. 2018). Yet, these same MAMPs/PAMPs can also mediate beneficial interactions with symbiotic microbes (Desaki et al. 2018). This broad surveillance system provides a significant barrier, but adapted bacteria have evolved sophisticated mechanisms to overcome it (Yu et al. 2019, Buscaill and van der Hoorn 2021, Wang et al. 2022).

In response to microbes that successfully bypass PTI, plants have developed a second, more specific line of defence. This is mediated by intracellular nucleotide-binding leucine-rich repeat (NLR) receptors that recognize specific pathogen effector proteins, leading to a robust defence response known as effector-triggered immunity (ETI). ETI often culminates in a hypersensitive response, which involves the death of infected cells to limit pathogen spread. This intricate, two-tiered immune architecture creates a perpetual cycle of adaptation and counter-adaptation, a classic evolutionary arms race between host and microbe (Saur et al. 2021, Chai et al. 2023). The broad pressure of PTI forces bacteria to evolve mechanisms to either mask their molecular patterns or suppress the general immune signalling, while the highly specific recognition of ETI drives the rapid diversification and modification of bacterial virulence factors.

### The evolutionary arms race for host colonization

#### Evolving evasion of host immune recognition

Both pathogenic and beneficial bacteria have evolved strategies to evade or modulate this immune surveillance. Beyond protein effectors (discussed below), bacteria employ chemical and structural tactics to interfere with plant defence signalling. Because PRRs detect highly specific epitope motifs, even subtle modifications in MAMPs can prevent or weaken recognition (Buscaill and van der Hoorn 2021).

**Figure 3:**
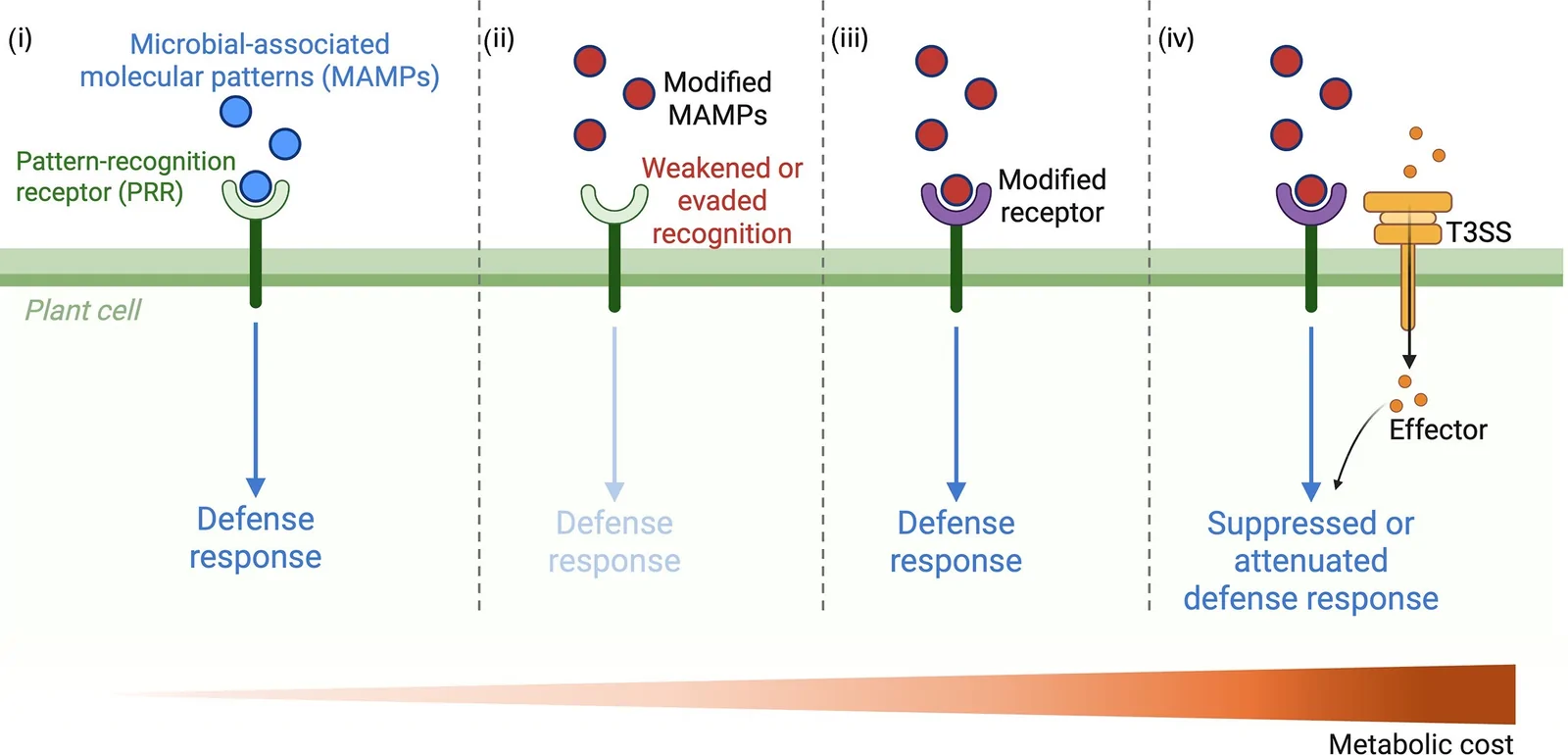
Conceptual model of co-evolutionary dynamics between plant immune recognition and bacterial immune evasion. From left to right, the figure illustrates *hypothetical* stages of the evolutionary arms race between plant innate immunity and plant-associated bacteria. This schematic representation does not refer to specific molecules but depicts generalized interactions to illustrate possible co-evolutionary trajectories. (i) Conserved microbial-associated molecular patterns (MAMPs) are perceived by plant pattern recognition receptors (PRRs), triggering PAMP-triggered immunity (PTI), and downstream defense responses. (ii) Bacterial populations evolve modifications in MAMP structure or presentation that reduce PRR recognition, resulting in attenuated or suppressed immune activation. (iii) In response, plants adapt by diversifying or refining PRRs, restoring recognition of the evolved MAMPs and re-establishing immune signalling. (iv) As a further counter-adaptation, bacteria deploy type III secretion system (T3SS)–delivered effector proteins that interfere with PRR-mediated signalling and downstream defense responses, effectively neutralizing host immunity. The gradient at the bottom reflects the increasing metabolic and regulatory cost associated with these successive adaptive innovations, reflecting trade-offs between immune evasion, growth efficiency, and ecological competitiveness. Image created partially with BioRender.com.

Structural modification or masking of MAMPs by some microbes serves as an adaptive mechanism to attenuate PTI (Fig. 3). For instance, symbiotic bacteria such as rhizobia possess structurally divergent LPS, exopolysaccharides, or peptidoglycan motifs that limit ROS production and promote root colonization (Downie 2010, Clúa et al. 2018). Pathogenic bacteria extend these strategies further, remodelling or degrading immunogenic molecules to escape host detection. *Pseudomonas* spp., for example, secrete the alkaline protease AprA, which cleaves monomeric flagellin peptides, the active elicitors of the FLS2 receptor, thus masking one of the most potent bacterial MAMPs (Bardoel et al. 2011). Other pathogens modify peptidoglycan through O-acetylation, produce lysozyme inhibitors, or deploy enzymes that suppress PGN-triggered responses, circumventing immune activation even after initial detection (Gust 2015, Long et al. 2018, Pinto et al. 2025). Notably, immune evasion does not always rely on direct modification or masking of immunogenic molecules. In the vascular pathogen *Xylella fastidiosa*, host adaptation has been linked to an evolutionarily reduced metabolic efficiency and an intrinsically slow growth phenotype. Genome-scale metabolic analyses indicate that this minimalist and fragile metabolic network constrains bacterial proliferation, supporting the view that fastidious growth functions as a self-limiting developmental strategy that maintains low population densities within the host and reduces immune detection (Gerlin et al. 2020). Crucially, these microbial strategies intersect with host-specific immune repertoires, such that the effectiveness of PTI evasion can vary between plant species: the flg15 peptide, a shortened derivative of the 22-amino acid flg22, is inactive in *Arabidopsis* or *Nicotiana benthamiana* but fully recognized in tomato, illustrating species-specific receptor recognition (Robatzek et al. 2007).

Plant receptor-like kinases (RLKs), including FLS2, EFR, and LysM-domain receptors, act as multifunctional hubs that mediate immune responses (Bhat and Haney 2025, Huang and Joosten 2025). Their evolution reflects a balance between stability and flexibility: they must reliably detect conserved microbial features while tolerating variation in microbial populations to avoid unnecessary immune activation. Although MAMPs tend to evolve slowly, subtle changes in flagellin, PGN, LPS, or other microbial motifs can modulate receptor binding and the strength of the immune response. This dynamic suggests that RLKs may experience adaptive pressures, refining recognition to prevalent microbial variants rather than evolving solely under purifying selection (elegantly discussed by Han 2019). This co-evolutionary landscape shapes both receptor diversity in plants and microbial strategies to evade detection, underpinning the establishment of both pathogenic and beneficial plant–microbe interactions (Fig. 3).

Overall, these structural and molecular evasive mechanisms represent the first evolutionary front in the plant–microbe interaction. When these fail, bacteria deploy specialized protein effectors that directly suppress host immunity, marking the next layer of the molecular arms race (Fig. 3).

#### The diversification of secretion systems and effector repertoires

A cornerstone of bacterial adaptation to plant hosts is the evolution of specialized protein secretion systems. Among them, T3SS represents a paradigmatic interface between bacteria and plant cells. This syringe-like nanomachine delivers a repertoire of effector proteins that mimic host molecules or interfere with key signalling pathways, thereby suppressing immunity and facilitating colonization (Deng et a. 2017 et al. 2017, Teulet et al. 2022, Bundalovic-Torma et al. 2022, Mesarich et al. 2015) (Fig. 3). In phytopathogens such as *P. syringae*, up to 16 effectors (Hop proteins) target plasmodesmata and defence signalling hubs (Li et al. 2021d). For instance, HopO1-1, bearing a ribosyltransferase domain, destabilizes plasmodesmata to promote bacterial spread (Aung et al. 2020), while AvrB activates the host MAPK MPK4, attenuating salicylic acid-mediated defence (Cui et al. 2010). Also, the type III effector HopAB1 (formerly AvrPtoB) in *P. syringae* functions to suppress host immunity, and HopI1 remodels thylakoid stacks within chloroplasts and suppresses salicylic acid mediated defences (Liu et al. 2022, Wang et al. 2023a, Liu et al. 2025). Similarly, *R. solanacearum* effector RipAC targets the ubiquitin ligase PUB4, destabilizing BIK1 and suppressing PTI signalling, while also interfering with ETI regulators such as SGT1 (Yu et al. 2020). The acquisition and diversification of these effector repertoires represent a major evolutionary leap, allowing for a highly targeted and sophisticated subversion of host defences. The continuous pressure exerted by host immune receptors drives the adaptive evolution of effectors, as pathogens constantly modify their proteins to evade recognition and to enhance virulence (McCann and Guttman 2008, Jwa et al. 2017). This dynamic is a prime example of an escalating arms race, where the pathogen’s gain of a new effector is met with the plant’s evolution of a new resistance gene, as discussed later.

However, this sophisticated molecular toolkit is not exclusive to pathogens. The T3SS is an ancient mechanism widespread among Gram-negative bacteria, including both pathogenic and symbiotic lineages. Its evolutionary plasticity is evident from the numerous independent losses and acquisitions of T3SS *loci* and effector repertoires documented in *Xanthomonas* species, which underpin transitions across a continuum of lifestyles from commensalism to pathogenicity (Pena et al. 2024). In beneficial associations such as the *Rhizobium–legume* symbiosis, the T3SS and its effectors have been co-opted to facilitate mutualistic interactions rather than disease (Miwa and Okazaki 2017, Teulet et al. 2022). Nitrogen-fixing rhizobia employ T3SS-delivered nodulation outer proteins (Nops) to modulate host immune responses and promote infection thread formation, thereby enabling root colonization. For instance, NopL from *Sinorhizobium* sp. NGR234 is phosphorylated by plant MAP kinases and acts as a decoy or inhibitor in defence signalling (Ge et al. 2016). Similarly, *Bradyrhizobium* employs T3 effectors such as ErnA and Sup3 to hijack the host SUMOylation pathway and induce nodules in *Aeschynomene* (Haq et al. 2025), whereas the effector SkP48 from another *Bradyrhizobium* strain encodes a SUMO protease that blocks nodulation in *Vigna radiata* and *Crotalaria juncea* (Piromyou et al. 2025). Mutations in those rhizobial T3SS or effector genes frequently result in poor nodulation, underscoring their central role in fine-tuning the immune equilibrium between host and symbiont.

#### Arms race dynamics vs. fluctuating selection: the balancing act of virulence

Bacterial pathogens frequently adjust their virulence programs in response to host-derived immune cues, illustrating the first step of counter-adaptation beyond simple immune evasion. A striking example comes from *R. solanacearum*, which modulates its nitric oxide (NO) detoxification machinery to withstand the oxidative and nitrosative burst induced by the plant immune system (Truchon et al. 2022). Notably, *R. solanacearum* has evolved an additional layer of sophistication: instead of merely neutralizing NO, it can exploit this host-derived molecule as a signal to activate its T3SS (Hendrich et al. 2023). This shift from mitigating host defences to repurposing them as regulatory cues, illustrates the escalation characteristic of host–pathogen coevolution and sets the stage for the broader dynamics described below.

Yet beyond such single-sided microbial adaptation, host–pathogen interactions often proceed through reciprocal changes, giving rise to co-evolutionary dynamics (Fig. 3). Antagonistic co-evolution between hosts and pathogens can manifest in different patterns: a continual escalation of traits known as *rrms race dynamics* (ARD) or a cyclical change in trait frequencies known as *fluctuating selection dynamics* (FSD) (Betts et al. 2014, Rafaluk-Mohr et al. 2022). These two patterns represent distinct forms of the so-called *Red Queen* dynamics (Brockhurst et al. 2014), which encompass both escalating and fluctuating evolutionary races between hosts and pathogens. ARD favours the evolution of a broader resistance range in the host and an increased host range in the pathogen, driving a directional selection for ever more potent virulence and resistance traits. A canonical example of ARD is the classic “gene-for-gene” co-evolution observed between the bacterial pathogen *P. syringae* and its host, *Arabidopsis thaliana*. In this system, the plant’s immune architecture illustrates how pathogens continually evolve effectors useful to suppress defence, while hosts counter-evolve layered surveillance strategies. For instance, the effector HopAB1 (AvrPtoB) targets the helper NLRs ADR1-L1 and ADR1-L2 to suppress immune signalling, yet *Arabidopsis* has evolved two complementary solutions: (i) sequence diversification in ADR1 prevents ubiquitination by HopAB1 (AvrPtoB), and (ii) the sensor NLR SNC1 “guards” ADR1-L1/L2 and activates ADR1 when suppression occurs (Wang et al. 2023b). This demonstrates how plants can deploy both diversification and multi-layered guard–guardee mechanisms to sustain resistance in the face of pathogen innovation.

However, this indefinite escalation is often tempered by a significant evolutionary constraint: fitness costs. The production of a new virulence effector or the constitutive activation of a resistance gene can be energetically expensive, imposing a cost on the organism in the absence of the specific co-evolving partner (Fig. 3). For instance, *RPM1* resistance gene in *A. thaliana* has been shown to incur a fitness penalty in the absence of the pathogen, resulting in a 9% decrease in seed production compared to plants lacking the gene (Tian et al. 2003). Comparable fitness costs also constrain pathogen evolution: in bacterial plant pathogens such as *R. solanacearum*, the production of key virulence determinants, notably exopolysaccharides, imposes a substantial metabolic burden that trades off against bacterial proliferation (Peyraud et al. 2016). This inherent trade-off can prevent the arms race from escalating indefinitely, pushing the co-evolutionary interaction toward FSD. In this dynamic, different pathogen genotypes are favoured at different times or in different host populations, maintaining genetic diversity within the species and preventing a single, “perfect” allele from sweeping the population (Lively et al. 1999, Han 2019). This balancing selection provides a compelling explanation for the persistence of avirulence alleles (pathogen genes recognized by host resistance genes) that would otherwise be purged from the population, as they may confer a fitness advantage in environments lacking the specific host resistance.

Importantly, these dynamics often play out at local or population scales, where demographic and ecological feedback are strong. At broader, biogeographical scales, however, the interplay among populations introduces additional layers of complexity, as described by *metapopulation theory* and the *geographical mosaic theory of coevolution* (Thompson 2005, Tao et al. 2024). Under this framework, coevolutionary “hotspots” and “coldspots” emerge across landscapes, reflecting heterogeneous selection pressures, gene flow, and ecological context. Thus, while fluctuating or escalatory *Red Queen* dynamics can dominate locally, the global coevolutionary landscape is best understood as a mosaic of interacting evolutionary trajectories.

### Molecular mimicry: the art of stealth and subversion

Molecular mimicry represents a highly sophisticated form of host manipulation that has evolved independently in diverse plant pathogens (Ronald and Joe, 2018). Rather than simply suppressing host defences, this strategy involves the production of molecules that functionally mimic endogenous plant signals, thereby co-opting the host’s own cellular communication network. A classic example is the phytotoxin coronatine, produced by *P. syringae*. This molecule acts as a structural and functional mimic of the plant hormone jasmonoyl-L-isoleucine (JA-Ile), and it binds to the plant’s JA receptor, activating the JA signalling pathway (Pieterse et al. 2012). In a classic display of crosstalk, this activation suppresses the salicylic acid mediated defence pathway, which is critical for fighting biotrophic pathogens, and ultimately promotes disease (Ishiga et al. 2010, Pieterse et al. 2012). The fact that pathogens have evolved distinct biosynthetic pathways to produce these mimics provides compelling evidence for convergent evolution, highlighting the central regulatory role of plant hormone signalling as a common evolutionary target.

Beyond small-molecule mimics such as coronatine, molecular mimicry can also occur at the protein level. Large-scale comparative analyses have identified a set of plant-resembling domains encoded by plant-associated microbes, termed PREPARADOs (plant-resembling plant-associated domains), which are enriched in bacterial genomes from plant-associated taxa and include domains previously described in effector proteins, such as ankyrin repeats, LRRs, or RCC1 (Levy et al. 2018). The presence of these domains suggests potential strategies by which bacteria could interact with plant cellular processes, for instance by engaging host signalling or immune-related pathways, although the functional roles of most PREPARADOs during plant colonization remain largely uncharacterized. In some cases, however, clear functional evidence for protein-based molecular mimicry exists. A well-characterized example is RaxX, a sulfated peptide produced by *Xanthomonas oryzae* pv. *oryzae*, which mimics plant PSY peptides involved in growth regulation. RaxX activates host growth responses while simultaneously being recognized by the XA21 immune receptor, illustrating how plants can evolve specific surveillance mechanisms against microbial mimics and highlighting plant peptide signalling as a recurrent evolutionary target (Pruitt et al. 2017).

## Co-metabolic integration and the evolution of cooperation in PAB

Once bacteria are well adapted to the plant environment, a range of symbiotic interactions can emerge, spanning a spectrum of associations where co-metabolism and cooperation often play key roles. Indeed, metabolic cooperation between plants and their associated bacteria is one of the most compelling examples of co-evolution across kingdoms. As mentioned above, plants and microbes are linked through the exchange of metabolites that act simultaneously as nutrients, signals, and regulators. Root exudates, including sugars, amino acids, organic acids, flavonoids, and phytohormone precursors, create a selective landscape that promotes bacterial specialization and niche differentiation. In return, bacteria transform, recycle, or detoxify these compounds, frequently generating metabolites that feed back into plant metabolism. Over evolutionary timescales, such reciprocal metabolic interactions have resulted in deep co-metabolic integration, where plant and bacterial pathways have become functionally complementary.

Within the rhizosphere, metabolic cooperation is not limited to nutrient exchange but extends to the fine regulation of plant stress physiology and growth signalling (Orozco-Mosqueda 2023 et al. 2023). A canonical example is the activity of bacterial 1-aminocyclopropane-1-carboxylate (ACC) deaminase, which cleaves the ethylene precursor ACC into α-ketobutyrate and ammonia (Glick 2014). This reaction prevents the accumulation of ethylene, a plant stress hormone that inhibits root elongation, and simultaneously supplies nitrogen to the bacterial partner (Glick 2014). Such dual functionality underscores the reciprocal metabolic logic of plant-microbe cooperation: the bacterium mitigates plant stress while exploiting a host-derived metabolite as a nutrient source.

Beneficial root-inhabiting microbes can hijack hormone-regulated immune signalling networks to establish a prolonged mutualistic relationship (Pieterse et al. 2012). A fascinating paradox in this co-evolutionary landscape is that some beneficial bacteria actively enhance plant immunity. Rather than evading or suppressing defences, these microbes can trigger a low-level, systemic immune response as a form of “protective inoculation”, creating a niche hostile to competitors (Conrath et al. 2006, Pieterse et al. 2014). This strategic modulation benefits the plant by priming its immune system against more aggressive pathogens, while simultaneously benefiting the microbe by reducing competition.

Interestingly, molecular parallels between bacterial and plant immune systems further reinforce this evolutionary continuum. For instance, the discovery of Toll/interleukin-1 receptor (TIR) domains in bacteria, homologous to those central in plant and animal immunity, suggests that components of immune signalling predate the divergence of these kingdoms (Lapin et al. 2022, Jia et al. 2023, Rousset et al. 2025). Together, such shared molecular strategies and signalling motifs underscore a deep evolutionary link between bacterial and plant immune systems, rooted in ancient mechanisms of cellular defence and communication

A paradigmatic case of evolved metabolic complementarity is the symbiosis between legumes and nitrogen-fixing rhizobia. The functional purpose of this association is a tightly regulated trade: the plant supplies reduced carbon compounds derived from photosynthesis, while the endosymbiotic bacteroids deliver fixed nitrogen in the form of ammonia (Lodwig and Poole 2003). Foundational studies in the 1960s demonstrated that *Bradyrhizobium japonicum* bacteroids oxidize C₄-dicarboxylates such as succinate and malate, confirming that these organic acids serve as key energy substrates driving N₂ fixation (Tuzimura and Meguro 1960, Yurgel and Kahn 2004). More recent genetic and physiological analyses indicate that the relative importance of individual C₄-dicarboxylates varies across symbioses, with L-malate often emerging as the predominant carbon source provided to bacteroids under physiological conditions (Mitsch et al. 2018). Transport of these substrates is largely mediated by dedicated systems, such as DctA/DctB/DctD, although alternative transporters like MaeP can provide functional redundancy in some strains, supporting substantial nitrogen fixation even in the absence of canonical Dct transport (Yurgel and Kahn 2004, Mitsch et al. 2018). This flexibility highlights the evolutionary plasticity of carbon acquisition strategies while maintaining the core principle of metabolic interdependence. Structural and functional refinement of transporter proteins toward selective recognition of C₄-dicarboxylates exemplifies how bacterial partners evolve to exploit host-derived nutrients efficiently, thereby stabilizing co-metabolic integration over evolutionary timescales (Yurgel and Kahn 2004, Mitsch et al. 2018).

Genome-scale metabolic modelling further illustrates how these symbioses evolve functional precision. In *Rhizobium leguminosarum* bv. *viciae* 3841, modelling predicted a strict reliance on dicarboxylates in the symbiotic state, but also revealed secondary carbon sources such as xylose and glycolate, corroborated by transcriptomic data (Schulte et al. 2022). Such models bridge molecular regulation and evolutionary adaptation, illuminating how energy flow becomes partitioned and optimized between partners.

The evolutionary depth of this co-metabolism is manifested in structural transformation. Within the plant cell, differentiated bacteroids reside inside the symbiosome—a transient organelle-like compartment specialized for nitrogen fixation (Udvardi and Day 1997). Host-derived nodule-specific cysteine-rich (NCR) peptides drive this differentiation, causing endoreduplication, morphological changes, and the loss of bacterial cell division capacity (Alunni and Gourion 2016). This process of terminal differentiation is an instance of symbiogenesis, where prolonged co-dependence can lead to irreversible integration (Meaney et al. 2020). Consequently, the bacterial genome undergoes reductive evolution: genes unnecessary for autonomous life, such as those for amino acid or cofactor biosynthesis, are lost (Meaney et al. 2020, Nikoh et al. 2014). Far from representing degeneration, this “genome decay” reflects adaptive specialization, in which complementary host functions compensate for lost pathways, solidifying the metabolic partnership.

An additional example of selective metabolic adaptation is provided by *Pseudomonas* species capable of salicylate degradation. Salicylic acid is a central plant phytohormone and defence signal (Tian et al. 2025). Certain *Pseudomonas* strains harbor the *sal* gene cluster enabling catabolism of substituted salicylates (Chakrabarty 1972, Christel et al. 2025). Yet, acquisition of this pathway by HGT does not necessarily confer an immediate fitness benefit. Experimental evidence shows that SA catabolism imposes minimal physiological cost but does not enhance bacterial fitness under tested rhizosphere conditions (Christel et al. 2025). Its ecological advantage may thus depend on specific community contexts or transient chemical conditions in which salicylate accumulation provides a selective niche. This illustrates a key evolutionary principle: HGT determines the rate of adaptation, while fluctuating host chemistry dictates the timing of selection. Hence, metabolic flexibility persists within the accessory genome as a reservoir of conditional fitness advantages that can be rapidly mobilized under specific ecological pressures.

Taken together, these examples reveal a continuum of co-metabolic evolution, ranging from facultative interactions based on opportunistic metabolite use to obligate symbioses that have undergone genomic integration. The recurring evolutionary pattern is that metabolic dependence, once stabilized by reciprocal benefit, leads to functional streamlining and genomic specialization. In this view, metabolic co-evolution can contribute to structural and genomic innovation in both partners.

However, while the molecular foundations of some interactions such as nitrogen fixation, ACC deaminase activity, or trace gas oxidation, are well established, many other proposed metabolic couplings remain hypothetical. The inference of evolutionary adaptation based solely on the presence of metabolic genes or ecological co-occurrence is insufficient. Demonstrating true co-metabolic integration requires quantitative validation of metabolite exchange, energy flow, and reciprocal fitness effects (Seto and Iwasa 2019, Pruss et al. 2023). The distinction between genomic potential and realized metabolic function is thus central to understanding how natural selection has shaped these interkingdom partnerships. Future work combining metabolic flux analysis, isotopic tracing, and synthetic community evolution will be essential to move from correlation to causation, revealing how deep functional complementarity emerges through evolution.

## Evolutionary dynamics at the community level

Microbial evolution in plant-associated environments extends beyond individual genomes to encompass the emergent properties of entire consortia (Vandenkoornhuyse et al. 2015, Rosenberg and Zilber-Rosenberg 2018, Hassani et al. 2018). Because plants and microbes experience shared selective forces, their interactions generate coupled ecological and evolutionary dynamics (Hacquard et al. 2015). The holobiont framework conceptualizes the plant and its microbiota as a single evolving entity (Vandenkoornhuyse et al. 2015) a perspective that has been further supported by both conceptual models and empirical studies of coevolution (Mesny et al. 2023).

Although this review focuses primarily on bacterial adaptation, understanding their evolution within plant-associated environments requires a community-level perspective, since bacterial fitness and innovation often depend on interactions with other taxa that collectively shape resource flows, signalling networks, and selective landscapes. Viewing the microbiome through this evolutionary lens provides the conceptual basis to understand how ecological interactions and environmental pressures shape adaptation and persistence at the community level.

### Community assembly: cooperation, competition and keystone taxa

The community coalescence framework provides a powerful perspective for understanding how previously distinct microbial consortia merge and reorganize, for instance, when roots recruit micro-organisms from bulk soil or when microbial inoculants are introduced into established communities. Each coalescence event can be viewed analogously to a selection process acting on collective traits such as cooperation, complementarity, and redundancy (Custer et al. 2024). Evidence from both natural and managed systems indicates that these interactions determine PAB community composition. In field microcosms, early colonizers with high network centrality act as keystone taxa that enhance diversity and predictability (Rawstern et al. 2025). Similarly, the establishment of bacterial inoculants depends on the pre-existing network structure and resource regime, with indirect interactions prevailing over direct competition (Garrido-Sanz et al. 2023).

Theoretical perspectives provide complementary explanations for the assembly of plant-associated microbiomes. Niche-based models highlight priority effects and deterministic filtering, whereas others emphasize context dependence and the importance of stochastic colonization events (Hawkes and Connor 2017). These frameworks converge on the prediction that ecological selection can favour functional complementarity and cooperation among coexisting taxa. Mechanistic studies support this view, showing that traits such as chemotaxis, quorum sensing, biofilm formation, and cross-kingdom interactions promote cooperative integration in the rhizosphere (Wang and Zou 2024, Appidi et al. 2025). However, the establishment of inoculant-driven communities remains strongly conditioned by their compatibility with resident consortia and by prevailing environmental conditions (Poppeliers et al. 2023). Thus, although cooperation can be a favoured evolutionary outcome, its actual expression is contingent on the ecological landscape in which microbial communities coalesce

Trophic regulation adds further complexity. In tomato rhizospheres, protist predation rewires microbial networks, expanding beneficial *Pseudomonas* and reducing pathogens (Song et al. 2024), demonstrating that top-down control can select for cooperative, disease-suppressive configurations. Furthermore, host phylogeny defines the interaction boundaries that constrain these processes (Fitzpatrick et al. 2018). Community assembly is therefore depicted as an evolutionary process shaped by network topology, trophic interactions, and host-mediated selection, in which management interventions can intentionally steer community evolution toward functional stability.

### External drivers of community evolution

Community evolution unfolds within broader environmental regimes that can reinforce, override, or redirect them. Abiotic and biotic stressors act as evolutionary filters that determine whether competition or cooperation dominates, shaping the selective environment in which communities evolve (Dastogeer et al. 2020). Global field experiments confirm this filtering effect, reporting consistent shifts in microbial diversity and convergence of community composition under nutrient enrichment and herbivore exclusion (Seabloom et al. 2023). It supports the view that common adaptive responses may operate across ecosystems.

Under global change, environmental forces generate eco-evolutionary feedback that couple plant physiology and microbial adaptation. Warming and altered precipitation simultaneously simplify microbial network architecture and reduce redundancy, thereby diminishing the buffering capacity of soil and plant-associated microbiomes (Hacquard et al. 2022, Li et al. 2024b). By contrast, persistent salinity promotes cooperation and functional specialization within halophytic microbiomes (Abdelfadil et al. 2024), suggesting that the duration and nature of stress dictate whether adaptation favours diversification or stabilization of mutualistic interactions. Consistent with this view, drought has been shown to induce highly conserved shifts in root-associated microbiomes, driven in part by plant-controlled changes in iron homeostasis that selectively enrich *Actinobacteria* in some plant systems (Xu et al. 2021).

Field evidence from post-disturbance systems further extends these patterns. Studies on soil and nodule microbiomes associated with *Alnus* species used for mine-soil reclamation show that shifts in soil pH and nitrogen availability filter microbial assemblages and reconfigure predicted metabolic pathways related to nitrogen fixation and organic carbon cycling (Thompson et al. 2025). Environmental rehabilitation may therefore function as an evolutionary sieve, favouring functionally redundant and stress-tolerant taxa that contribute to the recovery of ecosystem processes.

The combination of abiotic and biotic stressors exerts non-additive effects, with drought mitigating while warming amplifying pathogen pressure (Gallego-Tévar et al. 2024), showing that microbial evolution unfolds within multidimensional selective landscapes. Yet, not all filters act alike: acute, fluctuating stressors such as transient droughts often select for opportunistic, dispersal-prone taxa, whereas chronic pressures such as salinity, nutrient enrichment, or warming tend to stabilize cooperative guilds that invest in shared stress tolerance (Gallego-Tévar et al. 2024, Abdelfadil et al. 2024). Recognizing the temporal dimension of stress shifts our understanding of environmental filtering from a static process to a dynamic evolutionary force where the rate and predictability of disturbance determine the direction of community evolution.

### Agricultural legacies and domestication

Agricultural practices impose long-term directional selection on soil microbiomes, transforming management into an evolutionary force. Continuous monocropping reprograms both microbial diversity and host physiology (Li et al. 2019), while environmental filtering, whether driven by edaphic factors or management, acts as a deterministic process that constrains rhizosphere assembly and reduces stochasticity in community composition (Jing et al. 2022). Likewise, long-term agricultural intensification increases deterministic selection, reducing microbial diversity and altering community structure under conventional compared with organic farming (Hartmann et al. 2015). Viewed through an evolutionary perspective, management history may function as a heritable constraint on microbial evolution.

These long-term selective regimes leave measurable signatures at the community and functional level. Recent field studies clearly illustrate how different management intensities impose contrasting filters on rhizosphere assemblages. For instance, comparison among almond orchards under conventional, organic, and unmanaged regimes show that organic soils sustain higher fungal-to-bacterial ratios, greater functional guild diversity, and reduced pathogen abundance (Camacho-Sánchez et al. 2023), consistent with a shift toward disease-suppressive consortia and exemplifying how agricultural soils respond to contrasting selective regimes. Similarly, biocontrol inoculation reshapes rhizosphere assemblages and promotes beneficial functional groups (Rodríguez-Mena et al. 2022), illustrating that targeted interventions can modify established microbial networks. Management practices thus emerge as evolutionary drivers capable of redirecting community trajectories when compatible with resident consortia.

Intercropping—growing two or more crop species simultaneously—further exemplifies how management acts as a multi-layered selective force on the rhizosphere microbiome. A plethora of studies have shown that changing continuous monocropping to intercropping profoundly alters the plant-associated soil microbiome, with the changes heavily dependent on crop combination and intercropping duration (Domeignoz-Horta et al. 2024, Guo et al. 2025, Khashi u Rahman et al. 2025, Wang et al. 2024b). The mixed root exudates from different plant species act as a filter, selecting a unique microbial community combining both host-specific and generalist microbial taxa. For example, intercropping maize with legumes selects for specific microbes (e.g. *Bacillus, Pseudomonas*, and *Burkholderia* spp.) that are efficient phosphorus solubilizers (Gong et al. 2025, Zhang et al. 2025b). Recent studies show that certain bacteria evolve quickly, gaining new genes to use the different carbon sources from the component crops, thereby colonizing more than one species during intercropping (Gao and Zhang 2023, Sun et al. 2022, Xu et al. 2022). Successive cycles of intercropping drive the selection of these microbial assemblages, leading to the coevolution of the microbiome with the recurring plant community.

Crop rotation and domestication further shape the evolutionary memory of soils. Microbial similarity among crops reflects host phylogeny more strongly than agronomic performance (Kaplan et al. 2020), indicating that the ecological rationale for crop rotation may reside in disrupting microbial continuity associated with a particular plant lineage rather than merely alternating species. Breaking these microbial lineages helps prevent the accumulation of pathogens and rejuvenates functional diversity. In addition, breeding for high-input agriculture has narrowed endospheric diversity, reducing plant dependence on mutualistic partners (Compant et al. 2021), and reinforcing evolutionary path-dependence, where past selection constrains future adaptation. Under environmental stress (e.g. climate, salinity, pH, soil composition changes), these legacy effects become more pronounced, with communities shifting toward stress-tolerant but compositionally specialized assemblages (Li et al. 2024b, Abdelfadil et al. 2024). Thus, both rotation and domestication function as evolutionary filters that determine the adaptive potential of agricultural microbiomes.

### Host selection as an evolutionary stabilizer of core microbiota in plant holobionts

The host plant functions as an evolutionary architect of its microbiome. Root microbiomes mirror host phylogeny, establishing a macroevolutionary link between plant diversification and microbial recruitment (Fitzpatrick et al. 2018). This connection was later refined by showing that specific host *loci* modulate the abundance of functional bacterial groups (Tan et al. 2022), thereby linking genomic variation to microbial assembly. Beyond genetic determinants, host control operates spatially across plant compartments. Inside plant tissues, the endosphere imposes intense filtering, where only microbes compatible with host immunity and metabolism persist. In natural systems, host-specific recruitment patterns have been observed across contrasting plant lineages and ecosystems. In Iberian forests, blueberry and blackberry recruit distinct rhizosphere communities and differentially filter them into their endospheres, revealing consistent host-specific selection across compartments (Saati-Santamaría et al. 2023). Notably, despite this divergence, both species consistently enrich a shared subset of root-associated taxa in the endosphere, indicating the presence of compartment-specific recruitment signals that complement host divergence. Similarly, in the wild grass *Themeda triandra*, the rhizosphere and endosphere microbiomes conform to a two-step recruitment model dominated by deterministic host filtering and the emergence of a conserved endospheric core (Hodgson et al. 2025). Host-driven selection can therefore be regarded as a pervasive evolutionary mechanism structuring plant-associated microbiomes across environments and phylogenetic distances.

Host control, however, is not absolute. Reciprocal dynamics within the holobiont reveal that microbial communities are not passive passengers but semi-autonomous evolutionary entities whose internal interactions feed back on host selection and fitness (Hacquard et al. 2022, Mesny et al. 2023). In line with this idea, stabilizing effects can emerge from microbial interactions structured within the host environment, as shown in synthetic *Arabidopsis* root communities where auxin-degrading bacteria mitigate the root growth–inhibitory effects exerted by other community members, thereby buffering root system architecture through community-level interactions rather than direct host regulation (Finkel et al. 2020). Reconciling these host- and microbiome-centric perspectives requires acknowledging that selection acts simultaneously on both levels. Eco-evolutionary feedback couple these processes (Angulo et al. 2022): plant traits shape microbial communities, which in turn influence plant adaptative responses and the stability of the association (Kivlin et al. 2013). As a whole, host filtering and microbial autonomy may operate as intertwined selective forces driving the tempo and mode of plant-microbiome coevolution.

The balance between host control and microbial feedback is dynamically maintained by the metabolic and ecological processes that microbial communities exert on their hosts (Hacquard et al. 2022, Mesny et al. 2023). Patterns across compartments indicate that host metabolism not only filters microbial partners but also contributes to the stability and resilience of the whole consortium. Carbon released through roots, for instance, outweighs litter inputs in determining microbial composition, revealing that plants actively shape their microbial networks through carbon allocation (Feng et al. 2024). Beyond bulk carbon allocation, spatially and temporally restricted leakage of primary metabolites can also guide microbial assembly, as transient glutamine release from the root vasculature has been shown to create localized niches that attract and sustain specific bacterial populations (Tsai et al. 2025). This concept extends to the phyllosphere, where plant traits interact with climatic variables to coordinate above- and below-ground microbiomes (Wang et al. 2023a), suggesting that plants act as an ecosystem-level regulator that help maintain coherence among spatially separated microbial communities and stabilize their collective function under environmental variability. Consistent with this, defence phytohormone signalling plays a critical role in shaping root microbial communities, with disruption of these pathways leading to altered microbial profiles and reduced plant survival in natural soils. Moreover, salicylic acid can be differentially exploited by bacterial strains as either a growth signal or a carbon source, linking host immune regulation to dynamic microbial community restructuring (Lebeis et al. 2015). In addition, plant innate immunity maintains microbiome homeostasis by preventing opportunistic pathogens like *Xanthomonas* from causing tissue damage; loss of immune function leads to dysbiosis through pathogen-driven disruption of host tissues and consequent shifts in microbial communities (Pfeilmeier et al. 2024).

At evolutionary timescales, the host-microbiome partnership functions as a coupled adaptive system. Host immunity, metabolite exudation, and microbial adaptation are integrated into a model of reciprocal selection (Mesny et al. 2023), demonstrating that host performance depends on feedback within the holobiont. Direct physical interaction between plants and bacteria, rather than exposure to exudates alone, drives adaptive divergence among rhizosphere populations (Zhang et al. 2025c). Extending this concept, a conserved rhizosphere core enriched in nitrogen-metabolism genes has been identified (Cheng et al. 2024), suggesting that such core functions represent heritable extensions of plant functional traits conserved through coevolution. Metabolic coupling, reciprocal adaptation, and network-level integration may therefore contribute to the stability of the plant holobiont microbiome.

### Eco-evolutionary feedback and synthesis

Eco-evolutionary feedback capture the intertwined nature of ecological and evolutionary change in plant-microbiome systems. When shifts in community composition alter host physiology or resource flows, they generate new selective environments that, in turn, reshape microbial and plant traits. This recursive dynamic can transform short-term ecological responses into long-term evolutionary trajectories as shown by plant-microbe co-adaptation studies (Kivlin et al. 2013). Further recent work has shown that properties traditionally viewed as emergent, such as cooperation, redundancy or network connectivity, may themselves become targets of selection, as communities maintaining these traits persist and outperform others under environmental stress (Custer et al. 2024, Li et al. 2024b, Abdelfadil et al. 2024).

At the mechanistic level, multiple studies converge on the idea that evolutionary innovation is embedded within ecological networks. Nutrient gradients promote HGT, creating novel combinations of metabolic functions that facilitate adaptation to fluctuating conditions (Yang et al. 2024). Extending this reasoning to disturbance contexts, where pathogen invasion disrupts existing associations and redirects gene flow, such processes have been shown to produce new adaptive configurations (Xiao et al. 2024). It indicates that environmental variability does not simply erode community stability; instead, it can act as an evolutionary driver, favouring consortia capable of reconfiguring genetic and metabolic linkages. Conversely, excessive simplification of microbial networks under climate stress reduces redundancy and constrains this adaptive flexibility (Li et al. 2024b), while cross-kingdom linkages may contribute to network connectivity and cooperative stability (Wang and Zou 2024). The emerging picture is therefore one in which resilience and network integration may be linked, with systems with high network integration being more resistant to disturbance.

At the scale of the holobiont, these principles are expressed through reciprocal evolution between plants and their microbiota. Co-adapted *Streptomyces-*plant associations enhance drought tolerance through metabolic cooperation, exemplifying feedback where microbial evolution directly shapes host performance (Mesny et al. 2023, Liu et al. 2024c). Conceptually, such interactions have been framed within predictive models of eco-evolutionary dynamics (Angulo et al. 2022), suggesting that adaptation under global change depends on the coupling between ecological plasticity and evolutionary potential. These studies underscore the plant holobiont as a self-reinforcing adaptive unit, where feedback integrate metabolic, genetic, and community processes into a single evolutionary continuum.

Evidence from natural ecosystems underscores the generality of this framework. In arid biocrusts, cooperative consortia of cyanobacteria and actinobacteria maintain functional redundancy that underpins resilience and enables rapid recovery after desiccation (Miralles et al. 2023). These communities illustrate the same principles of feedback-driven stability observed in plant microbiomes: diversity begets cooperation, and cooperation sustains adaptation. Such parallels suggest that eco-evolutionary coupling may represent a widespread property of microbial collectives, independent of host association.

Recognizing microbial communities as evolving entities rather than static assemblages fundamentally alters how we approach microbiome management. If traits such as cooperation, redundancy, and connectivity are selectable, they can be intentionally fostered through agricultural and restoration practices. From this perspective, microbiome engineering becomes an exercise in guiding evolutionary trajectories, aligning ecological interventions with long-term adaptive outcomes. Integrating eco-evolutionary feedback into predictive models thus provides not only a conceptual synthesis but also a roadmap for designing resilient microbial ecosystems capable of sustaining plant productivity under accelerating environmental change.

## Conclusion

Bacterial adaptation to plant environments arises from the integration of genome plasticity, regulatory complexity, and ecological filtering. HGT, regulatory fine-tuning, and metabolic specialization collectively facilitate the exploitation of plant-derived substrates, the coordination of motility and adhesion, and the modulation or evasion of plant immunity. Together, these mechanisms underpin the remarkable diversity of plant-associated lifestyles, from beneficial symbioses to pathogenic interactions.

Despite substantial progress, our understanding of how these processes operate in natural contexts remains incomplete. The relative contributions of selection and drift across plant compartments, the conditions under which horizontally acquired traits become adaptive, and the functional implications of regulatory rewiring are still poorly resolved. Moreover, the field remains dominated by a limited number of model systems (e.g. rhizobia, *Pseudomonas, R. solanacearum*), whereas many PAB and interaction types are insufficiently explored. Integrating perspectives from evolutionary biology, microbial ecology, and plant sciences remains challenging due to differences in scale, assumptions, and methodology, yet is essential for a unified understanding of plant-microbe evolution.

Key challenges ahead include elucidating how bacteria integrate multiple host- and environment-derived cues to regulate colonization, competition, and cooperation within plant tissues. Advances in single-cell, spatial, and high-resolution techniques will be crucial to link molecular mechanisms to emergent behaviours at population and community levels, and to clarify how microbial assemblages establish, persist, and influence plant fitness across heterogeneous environments.

Future work should also address the ecological relevance of accessory genomic and metabolic diversity, quantify the contexts in which these traits confer selective advantages, and determine how community processes shape evolutionary trajectories in plant microbiomes. Such efforts will help distinguish stable adaptive features from transient or conditionally beneficial traits.

By combining multidisciplinary approaches with rigorous empirical frameworks, the field is poised to move from descriptive microbiome characterizations toward predictive, evolution-informed models of plant-microbe interactions. These advances will refine our understanding of holobiont evolution and support the rational design of microbial strategies for sustainable agriculture.
